# Promoter trapping method: transcription factor purification using human telomerase reverse transcriptase promoter

**DOI:** 10.1186/s12953-014-0053-2

**Published:** 2014-11-18

**Authors:** Linda I Nagore, YanWen Zhou, Robert J Nadeau, YinShan Jia, Harry W Jarrett

**Affiliations:** Department of Chemistry, University of Texas at San Antonio, One UTSA Circle, San Antonio, TX 78249 USA

**Keywords:** Mass spectrometry, Transcription factor, Chromatography, Proteomics, Purification

## Abstract

**Background:**

Transcription factors bind to response elements on the promoter regions of genes to regulate transcriptional activity. One of the major problems with identifying transcription factors is their low abundance relative to other proteins in the cell. Developing a purification technique specific for transcription factors is crucial to the understanding of gene regulation. Promoter trapping is a method developed that uses the promoter regions as bait to trap proteins of interest and then purified using column chromatography. Here we utilize this technique to study the telomerase promoter, which has increased transcriptional activity in cancer cells. Gaining insight on how to control the enzyme at the promoter level may give new routes towards cancer treatments.

**Results:**

Our findings show that the telomerase promoter (−170 - +91) and Promoter Trapping isolate a transcriptionally active and reproducible complex, when analyzed by liquid chromatography tandem mass spectrometry. We were also able to identify transcription factors, including AP-2 and SP1 known to bind this promoter, as well as show that these two proteins can bind to each other’s response element.

**Conclusion:**

Here we focus on verifying the ability and versatility of Promoter Trapping coupled with additional well-characterized methods to identify already known factors responsible for telomerase transcriptional regulation.

**Electronic supplementary material:**

The online version of this article (doi:10.1186/s12953-014-0053-2) contains supplementary material, which is available to authorized users.

## Background

In most human somatic cells, human telomerase reverse transcriptase (hTERT) activity is silenced or present at very low levels whereas cancer cells, germ line cells, and embryonic stem cells have elevated hTERT activity [1,2]. hTERT is an enzyme that adds repeats of a guanine rich sequence, called telomeres, to the ends of chromatids [3,4]. Telomeres are DNA-protein complexes that shield the ends of chromosomes from degradation and fusion by creating a protective cap [5]. At birth telomeres are approximately 15 kb long and after 50 to 70 cell divisions the telomeres undergoes a progressive shortening, making them too short for replication leading to cell senescence and eventually death [5,6]. However, it has been found that in 90% of malignant cells hTERT activity is increased causing the cell’s telomeres to regenerate and the cells become immortal [7]. The transcriptional regulation of hTERT is the subject of a recent review [8].

**Figure 1:**
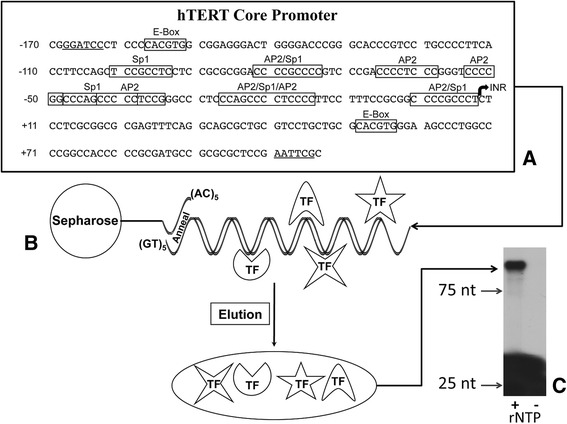
**Promoter trapping with the hTERT promoter yields a transcriptionally active complex. A**. Nucleotide sequence of the hTERT gene regulatory region. Features of known binding regions are noted, such as two E-Boxes, AP2, and SP1 sites. The translational start site (INR), nucleotide +1, is denoted by the bold arrow. **B**. Full hTERT promoter containing single stranded (GT)_5_ tails complexed with TF from HEK293 NE. hTERT specific TF are purified by annealing (GT)_5_ tails to a (CA)_5_-Sepharose column and eluted with a high salt buffer. **C**. Transcription Assay of hTERT Promoter Trap Elute. After RNA was transcribed by the promoter trapping eluate, 0.1 pmol ^32^P-labeled oligo (5′-cggagcgcgcggcatcgcgg-3′) was extended with reverse transcriptase and analyzed on a 6% polyacrylamide gel containing 8 M urea. Visualization was accomplished with autoradiography. (+) Indicates the use of rNTP and (−) denotes where rNTP was not used during transcription, as a negative control. The expected product is 100 base pairs.

There are several transcription factor (TF) binding sites on the hTERT promoter, shown in Figure 1. For example, specificity protein (SP1), Enhancer Box (E-Box) binding TFs and activator protein 2 (AP-2) are all TFs known to bind the hTERT promoter. Sp1, a C2H2-type zinc-finger protein that binds to GC-rich motifs, contains five binding sites within the promoter sequence and has been found to stimulate or suppress transcription depending upon its post-translational modifications. AP-2 binds to the GCCNNNGGC consensus sequence and has been found to have seven binding sites on the hTERT promoter. Many of the SP1 and AP-2 binding sites overlap and we show here a strong influence on each other.

Transcription of hTERT is regulated by TFs, which activate or repress expression. Despite the importance of the hTERT gene on cell growth, longevity, and tumor formation, little is known about how it is regulated at the transcriptional level. *In silico* methods identify several potential TFs, however, experimental verification is often lacking. Most promoter analysis has consisted of the identification of a single TF bound to this promoter, at a given time, and under given conditions. These experiments are laborious and fail to identify the complete set of TFs bound to a particular promoter. A method, called Promoter Trapping [9], has been developed where the promoter is “tailed” with single stranded (GT)_5_. The DNA-protein complex is allowed to form in solution. The promoter is then annealed to (CA)_5_-Sepharose and any irrelevant proteins can be washed away and the bound proteins eluted.

Previously, we had developed promoter trapping using the c-jun promoter, which has very high promoter activity in reporter assays. Here, promoter trapping [9] was performed using nuclear extract (NE) from the HEK293 cell line using the hTERT promoter. An overview of the workflow can be seen in Figure 1. Reporter assays show this promoter to be 4500-fold less active than the c-jun promoter and yet promoter trapping results in a transcriptionally active transcription complex. The complex was characterized by Western and Southwestern blots and using LC-MS/MS. This characterization of the complex reveals it to be very reproducible and to contain not only many of the proteins of the RNA polymerase 2 general transcription machinery but also specific transcription factors (SP1, AP2, and USF2) known to bind this promoter. Thus, promoter trapping is a highly reproducible method that can be applied to promoters over a wide-range of promoter strengths.

## Results and conclusion

Promoter trapping (PT) is a method that utilizes DNA response elements present in a gene’s promoter region (100–1000 base pairs) to enrich for factors responsible for gene regulation. This method has been used to successfully purify the transcription complex bound to the c-jun promoter [9]. To extend this method to other promoters, we applied this technique in the purification of hTERT-specific TFs as well as general components of transcription by using the hTERT core promoter.

Figure 1 depicts the PT method along with the core promoter sequence used and some of its known binding sites. To demonstrate the validity of this method and its ability to purify TFs from any promoter we focus on known TFs that bind to the hTERT promoter. Not only does this method enrich for specific low abundant proteins but also it is able to capture a functional transcription complex, which was confirmed with a transcription assay (Figure 1C). Here, the promoter was transcribed to RNA, isolated, and then reverse transcribed using specific primer oligonucleotides. In order for transcription to occur a number of factors must be present, one being rNTPs. Thus, by the removal of rNTPs confirms that any bands visualized from the assay are exclusive to transcription. PT eluate with rNTP (+) and PT eluate without rNTP (−) were assessed side by side and as expected the (+) lane produced a well-defined band of the expected size demonstrating that the active transcription complex was isolated following promoter trapping while the (−) was blank. An additional negative control was utilized which included the reaction mixture minus hTERT promoter DNA, and also produced a null result (data not shown).

**Figure 2:**
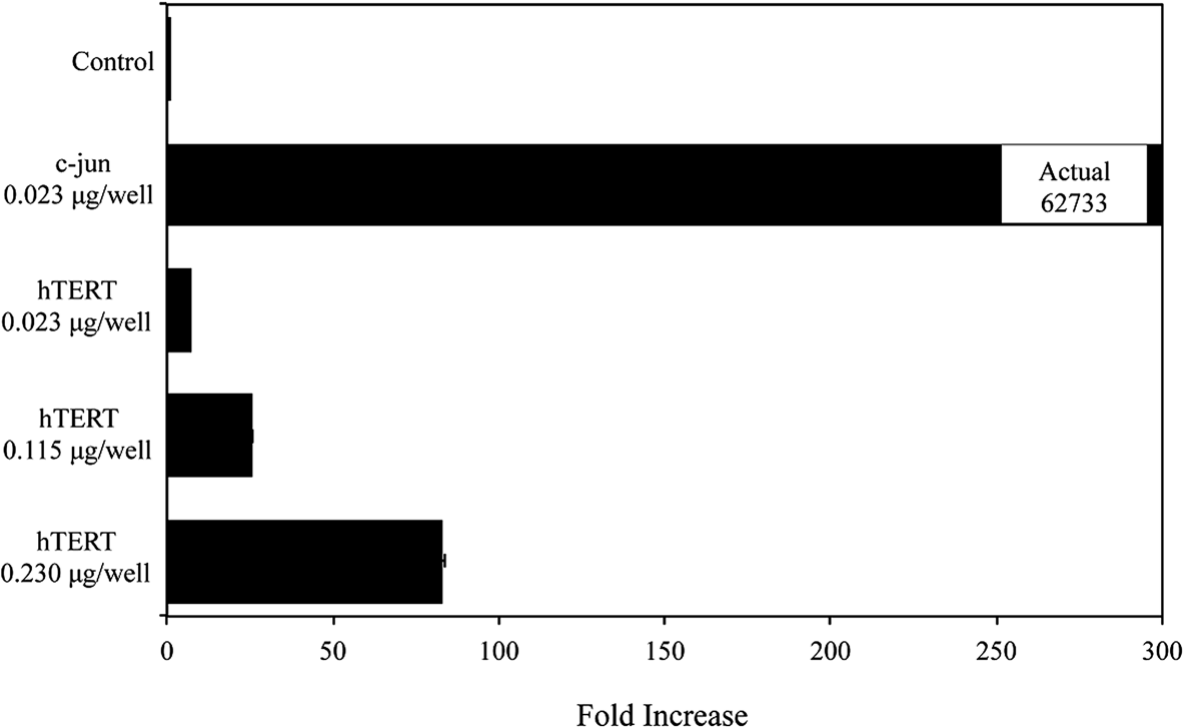
**Analysis of hTERT promoter activity using dual luciferase assay.** hTERT was subcloned from hTERT-pUC19 into pMLUC. During transfection, pTK-LUC and hTERT-pMLUC or the empty vector control were mixed and transfected into cell line HEK293. Cells were lysed 48 hours later and acitivity was measured with a dual luciferase assay. Two measurements were taken, renilla and firefly luciferase, and the ratio of the two was used to measure the activity of each condition. The average of triplicates (from top to bottom) were 1.9, 62733, 13.7, 50, and 160.6. cJun is 4579 times higher activity than hTERT.

The promoter complex’s activity *in vivo* is shown with a reporter assay in Figure 2. The empty vector negative control shows basal activity, while the hTERT promoter construct demonstrates a dose-dependent activity. This experiment was executed next to the c-jun promoter to show the relative activity. The hTERT promoter has a lower endogenous activity in HEK293 cells when compared to the c-jun promoter, which gave a 4500-fold lower signal at the same dose. Thus, even with a much less active promoter, promoter trapping yields a transcriptionally active complex.

**Figure 3:**
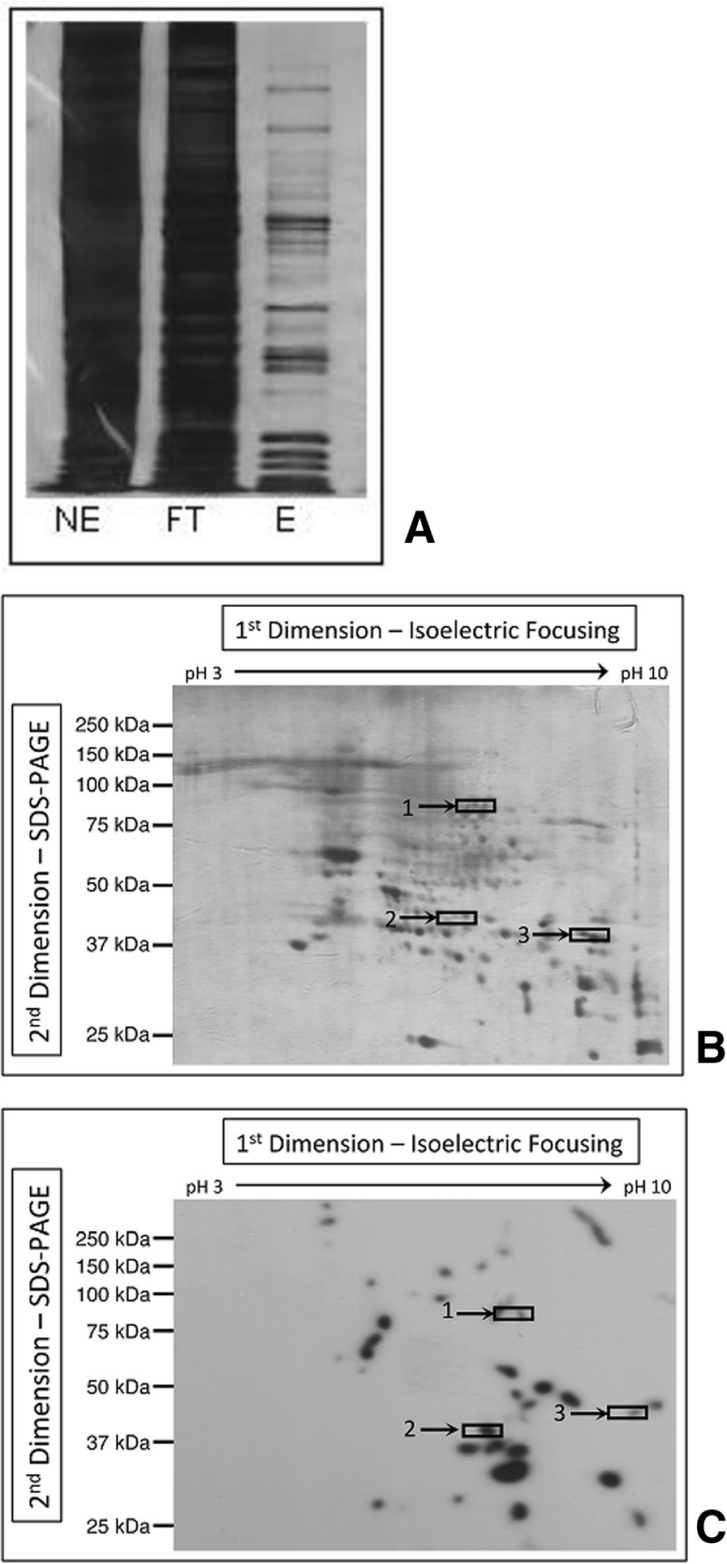
**Analysis of hTERT TF purification by gel electrophoresis and Southwestern blotting. (A)** Silver stained 1D Gel Electrophoresis using hTERT promoter trapping. Proteins from the eluate were separated on a 7.5% SDS polyacrylamide gel. Lanes consist of NE, flow through (FT), and elute (E). **(B)** Two-Dimensional PAGE. Eluate was subjected to 2DGE gel electrophoresis. The first dimension, isoelectric focusing, was achieved with a 7 cm, pH 3–10 IPG strip. The second dimension, separation by molecular weight, was done with 12% sodium dodecylsulphate-polyacrylamide gel electrophoresis (SDS-PAGE). Visualization was accomplished by silver staining. **(C)** DNA-Binding by Two-Dimensional Southwestern Blot. The 2-DE gel shown in Figure 1 was electroblotted onto a PVDF membrane and probed with the 2.0 nM radiolabeled hTERT promoter. Spots indicate the number of high affinity DNA-binding components of the hTERT complex that were purified by promoter trapping. In panels **B** and **C**, boxes with numbers show regions of the blot excised for further analysis.

The extent of the purification using the promoter trapping method can be seen in Figure 3A. The first lane demonstrates the complexity of nuclear extract (NE) with the multitude of bands present. The flow through (FT) has a similar pattern with similar intensity as the NE, indicative of the low abundance of promoter specific proteins involved in regulating a single promoter. The washes were also collected (data not shown) and were equally complex as the NE and FT. The DNA-protein complex was eluted with 0.5 M NaCl (E), which disrupts the DNA-protein binding, allowing proteins to elute while the DNA remains on the column. The eluate displayed a much simpler protein mixture, as seen by silver staining, though it still contained many components, as seen in Figure 3B. The many components were further resolved by two-dimensional gel electrophoresis (2DGE) (Figure 3B) and silver stained for protein visualization. Although the sample was purified using the PT method, the eluate is made up of protein-binding proteins and DNA-binding proteins. While the non-DNA binding proteins are not TFs they are still significant since they are involved in transcriptional regulation, however we will focus on the DNA-binding components. In order to identify how many proteins are specifically DNA-binding proteins a two-dimensional southwestern blot (2DGE-SW) was prepared and probed with 2 nM hTERT (Figure 3C). Not only does the southwestern blot give information on the number of DNA-binding proteins involved along with the molecular weights and their respective pI (shown in Figure 3C) but it can also be used as a tool to study transcriptional regulation [10]. The comparison of the 2DGE-SW (Figure 3C) to the protein stained 2DGE (Figure 3B) shows that there are significantly less spots in the 2DGE-SW, showing that the DNA-binding proteins are enriched as well as these other protein that do not bind DNA. A number of spots in Figure 3C have physical properties similar to the transcription factors known to bind to the hTERT promoter such as specificity protein (SP1, MW = 97 kDa, pI = 6.9), TATA binding protein (TBP, MW = 38 kDa, pI = 9.8) and upstream stimulatory factor (USF-2, MW = 44 kDa, pI ~ 5).

**Figure 4:**
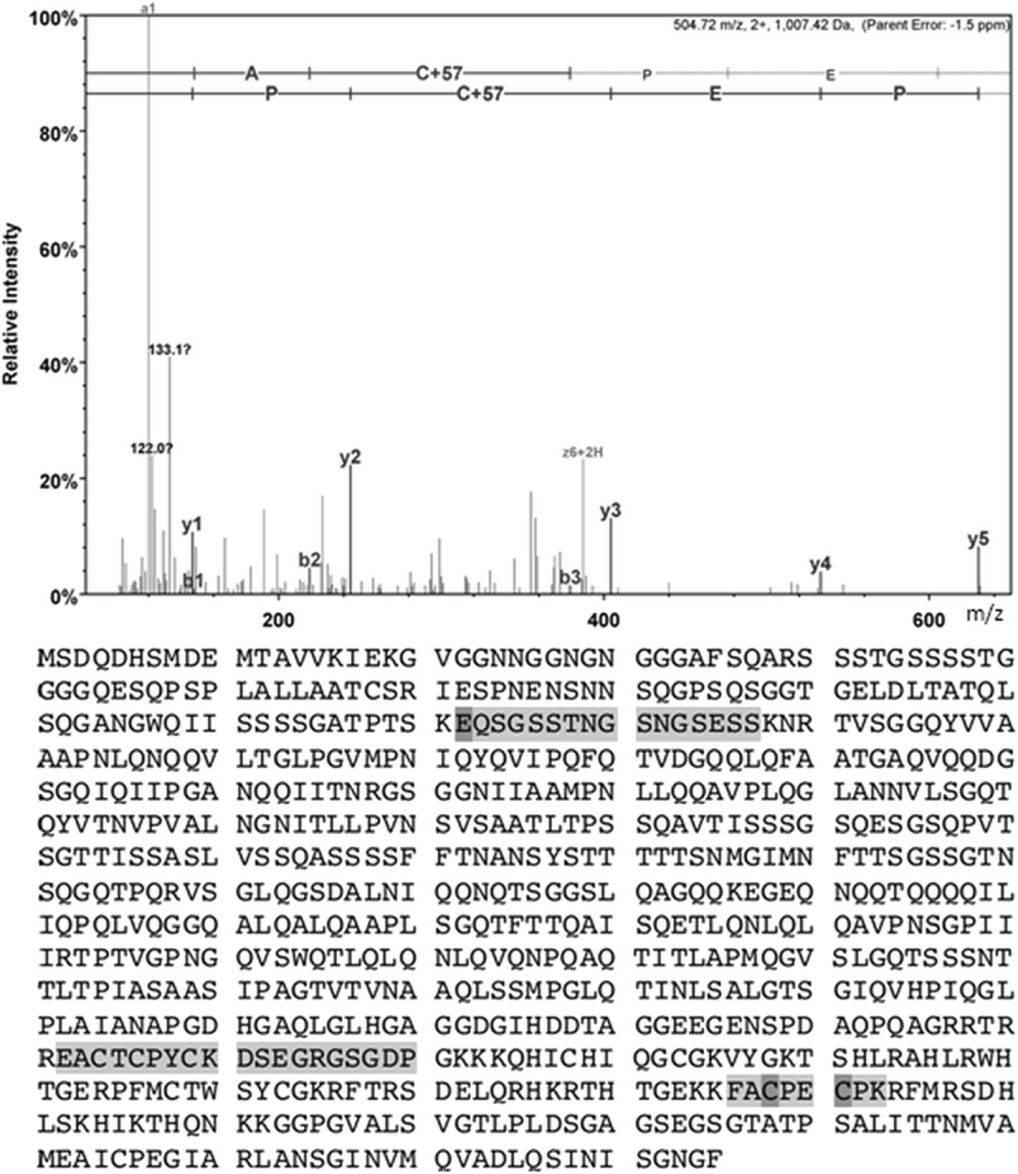
**MS/MS fragmentation of FA** ***C*** **PE** ***C*** **PK found in SP1_HUMAN.** Transcription factor specificity protein 1 (SP1) was identified by LC-MS/MS and was matched to 504.7168 m/z (2+) by protein database searching with Mascot software utilizing the SwissProt database. SP1 was identified with a Mascot protein score of 33 and sequence coverage of 5%. The top-ranked tryptic peptide from SP1 contained amino acid **FA** ***C*** **PE** ***C*** **PK**, spanning amino acid residues 686–693 with an expectation value of 0.0056. The italicized amino acids indicate that they are carbamidomethyl modified cysteines.

**Figure 5:**
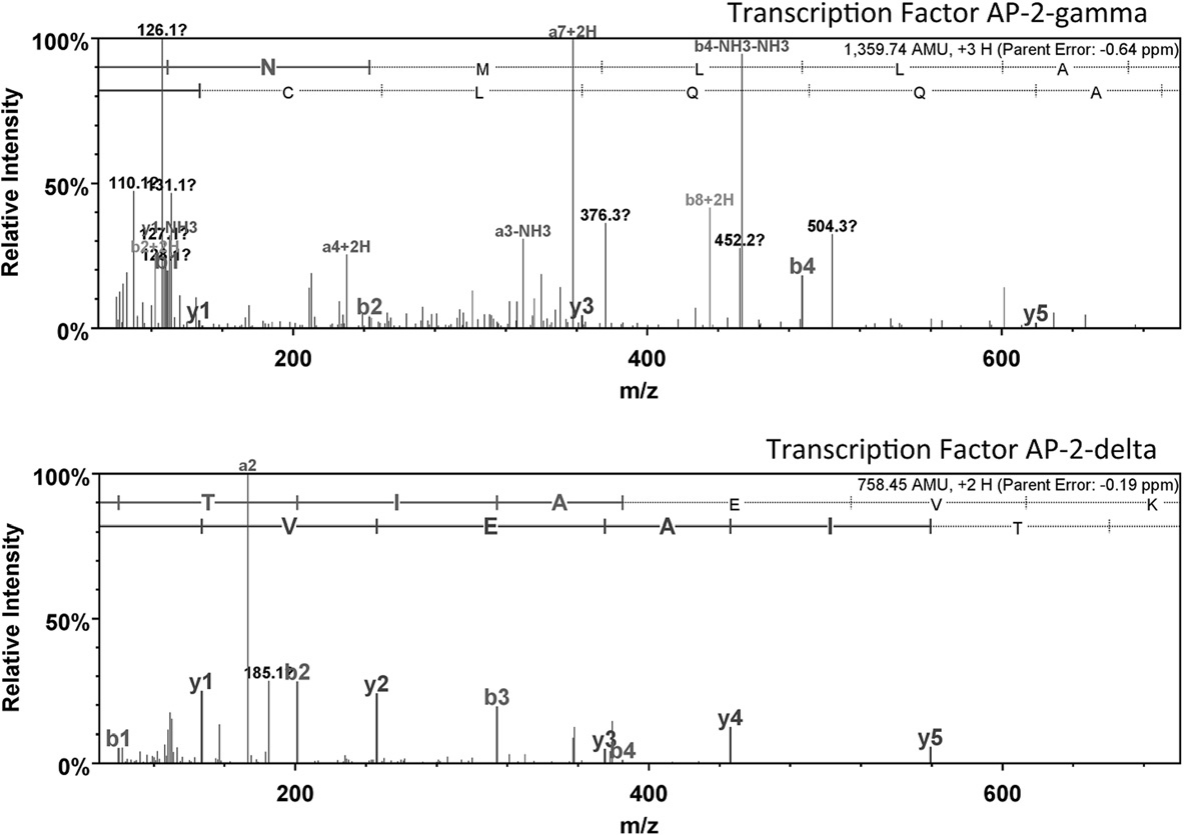
**MS/MS fragmentation of peptides corresponding to AP2C_HUMAN and AP2D_HUMAN.** Transcription factor activator protein 2 (AP-2) was identified by LC/MSMS and was matched to two different isoforms. AP-2-gamma (AP2C) was identified from m/z = 454.25 (3+) and AP-2-delta (AP2D) from m/z = 380.23 (2+) by protein database searching with Mascot software utilizing the SwissProt database. AP2C was identified with a Mascot protein score of 25 and sequence coverage of 3% and 45% probability while AP2D was identified with a Mascot protein score of 33.5 and 4% sequence coverage and 89% probability. The top-ranked tryptic peptide from AP2D contained amino acid **VTIAEVK**, spanning amino acid residues 229–235 with an expectation value of 0.002. The top-ranked tryptic peptide from AP2C contained amino acid **KNMLLAAQQLCK**, spanning amino acid residues 349–360 with an expectation value of 0.0007.

**Figure 6:**
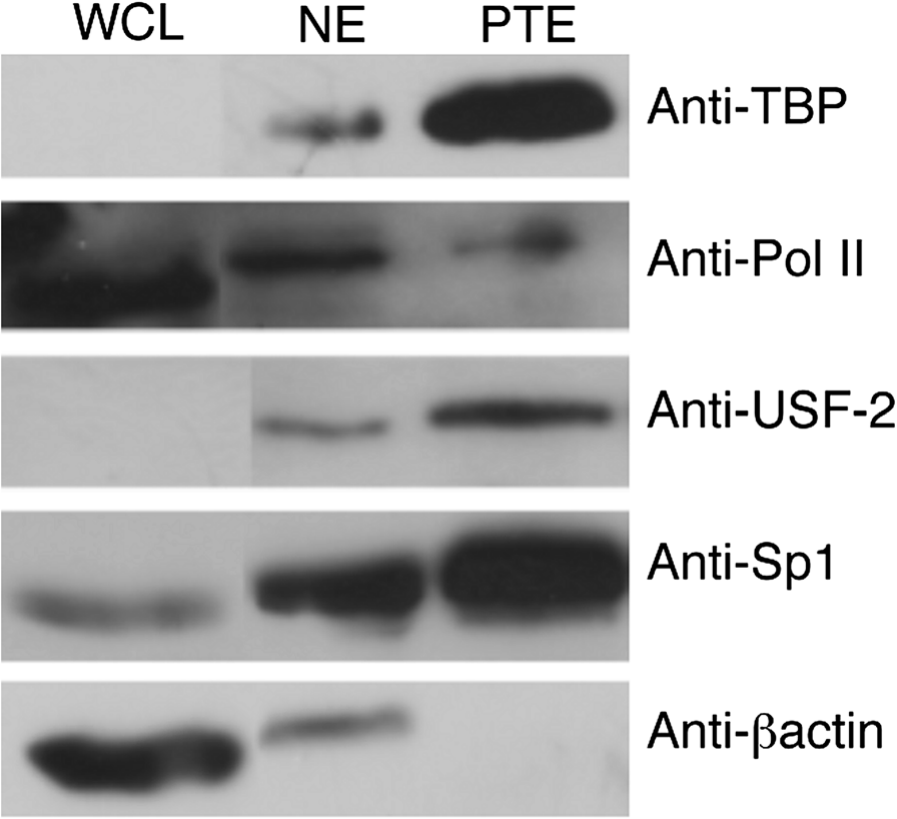
**Transcription characterization with 1-D WB.** TF characterization with one-dimensional Western blotting (1-D WB). 1D-SDS-PAGE gel was electro-blotted onto a PVDF membrane and then probed with the following antibodies: TATA binding protein (TBP), RNA Polymerase II (Pol II), upstream stimulatory factor 2 (USF2), specificity protein 1 (SP1), and βactin. The individual lanes of the SDS-PAGE gel were loaded with whole cell lysate (WCL, protein from the cytosol as well as nucleus) obtained from sonication followed by centrifugation to remove cell debris; nuclear extract (NE); hTERT promoter trapping eluent (PTE). Bands indicate components that are specific to the hTERT promoter as well as demonstrate purification of specific TF from the complex mixtures of WCL and NE.

Mass spectrometry is a useful tool for protein characterization and identification especially when combined with purification techniques such as PT and 2DGE. Gel plugs believed to represent TFs, based on their physical properties and bands with a darker appearance in the 2DSW were excised from the 2DGE. The excised proteins were then digested, extracted from the gel plug, and the peptides were separated on a C-18 column. SP1 and AP2 were confirmed as presented in Figures 4 and 5, respectively. While all identifications are statistically significant, the sequence coverage of each of the specific TFs were below our normal benchmark; however, with MS/MS sequencing producing expected values below 0.005 and the supporting evidence from the Western (Figure 6) and Southwestern blots (Figure 3C) confirm the results are significant.

A repressor known to be involved in hTERT regulation is transcriptional repressor CTCF (TR-CTCF). We were able to purify and identify TR-CTCF through PT-MS/MS with 96% probability (data not shown). Other hTERT specific TF were also found including p53, TGF-β, as well as proteins from the Mad and STAT families (data not shown).

**Figure 7:**
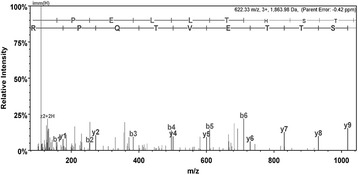
**MS/MS fragmentation of RPELLTHSTTEVTQPR found in GTF2-I_HUMAN.** General transcription factor II-I (TFII-I) was identified by LC/MSMS and was matched to 622.3338 m/z (3+) by protein database searching with Mascot software utilizing the SwissProt database. TFII-II was identified with a Mascot protein score of 77 and sequence coverage of 6%. The top-ranked tryptic peptide from TFII-I contained amino acid RPELLTHSTTEVTQPR, spanning amino acid residues 540–555 with an expectation value of 5.9 × 10^−05^.

While we have discussed hTERT specific factors there are also general TF that are important to the transcriptional machinery. One of the general TFs with the highest identification score involved in transcriptional complexes is General Transcription Factor II-I (GTF2-I) with an expectation value of 5.9 × 10^−05^ (shown in Figure 7). GTF2-I has been known to co-regulate hTERT activity with USF (Upstream Stimulatory Factor) [11], which was also shown to be present in the promoter complex (Figure 6) [12]. The higher abundance of general transcription factors following promoter trapping allows isolation and identification by mass spectrometry as well as the specific TFs such as AP2 and SP1.

Western blots were not only used to verify the presence of TF but also show the extent of TF enrichment by PT (Figure 6). Whole cell lysate (WCL), nuclear extract (NE), and the eluate from promoter trapping (PTE) were probed with five different antibodies to illustrate the enrichment capabilities of PT. Sp1, USF-2 and TBP are clearly enriched in the PTE relative to NE. These results are perhaps not surprising since there are five recognition sites within the hTERT promoter for Sp1 and two sites that potentially bind USF-2, allowing their enrichment by PT. RNA Polymerase II (Pol II) had a similar result, although not as strong of a band as NE; it is reasonable that the nucleus contains excess Pol II. β-actin, an abundant cellular protein, is not enriched by PT and provides a negative control.

**Figure 8:**
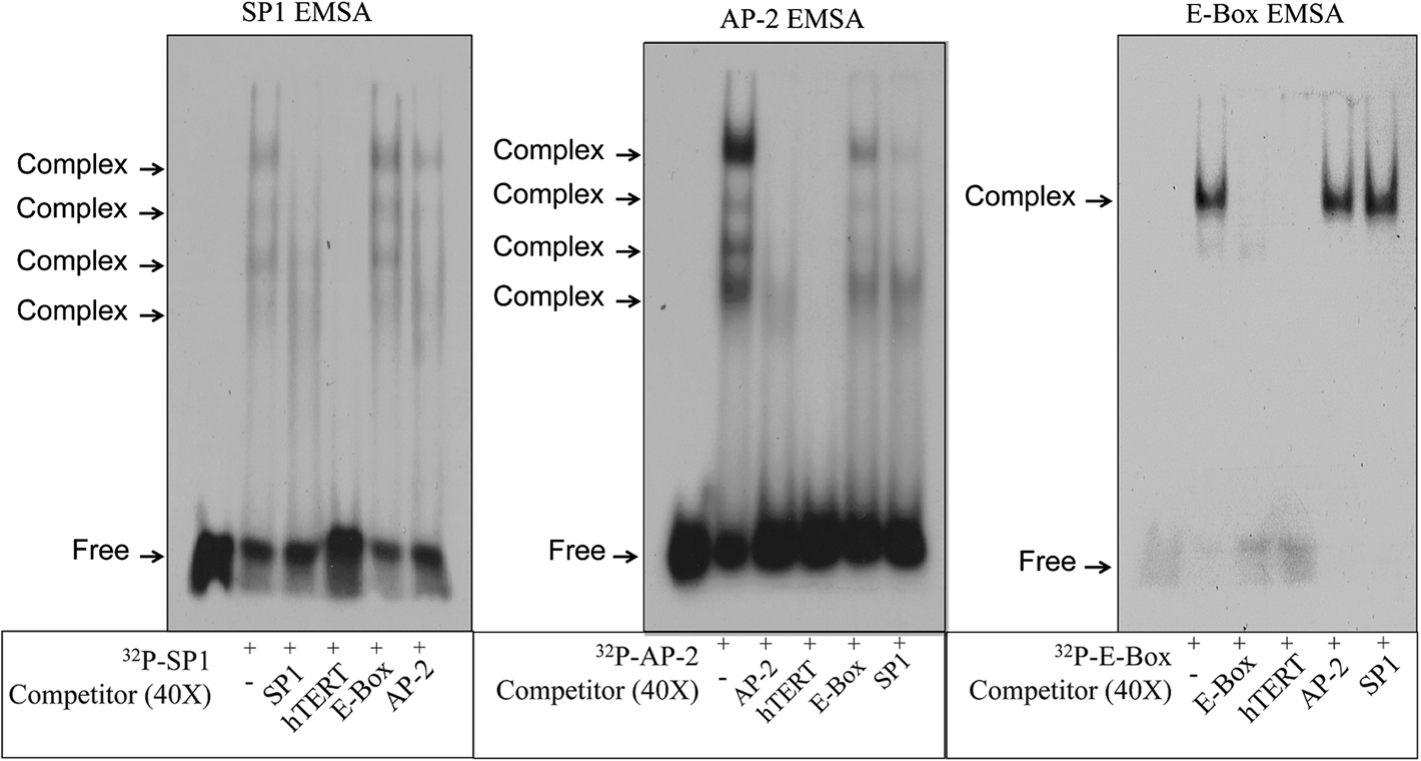
**Electrophoretic Mobility Shift Assay (EMSA) of** ^**32**^ **P-labeled DNA containing specific DNA sites to study DNA-protein interactions.** From left to right, radiolabeled SP1, AP-2 and E-Box specific oligonucleotides were incubated with promoter trap eluate. Competitor DNA (unlabeled) was added to show the specificity for hTERT promoter DNA as well as interactions.

A competitive gel-shift experiment (Figure 8) was designed using transcription factors known to have interactions with hTERT and canonical binding site oligonucleotides. The three frames show gel shifts of radiolabeled SP1, AP-2, and the E-box oligonucleotides, respectively. The binding was competed with a 40x excess of unlabeled oligonucleotides or DNA. When the complete hTERT promoter DNA is used as the competitor, the shifted bands are diminished in all experiments showing this contains similar DNA sequences to the canonical oligonucleotides used. To determine if the overlap of SP1 and AP-2 sites shown in Figure 1 has a functional significance, each oligonucleotide was used for both the gel shift and as a competitor. Clearly, each oligonucleotide competes with the other while neither competes for the E-box gel shift. When ^32^P-SP1 is competed with the unlabeled AP-2 oligonucleotide or ^32^P-AP-2 competes with unlabeled SP1, certain bands are diminished. This suggests that AP-2 and SP1 not only interacts with hTERT promoter but also compete with each other’s binding. Evidence of TF interacting with each other within the same promoter has been previously identified on the PAI-1 gene [13]. This competition suggests that either transcription factor can bind to the other’s consensus DNA sequence or there is some protein-protein interaction between the two. The E-box gel shift demonstrates a simpler case where there is only competition with the E-box oligonucleotide and the complete hTERT promoter sequence.

**Figure 9:**
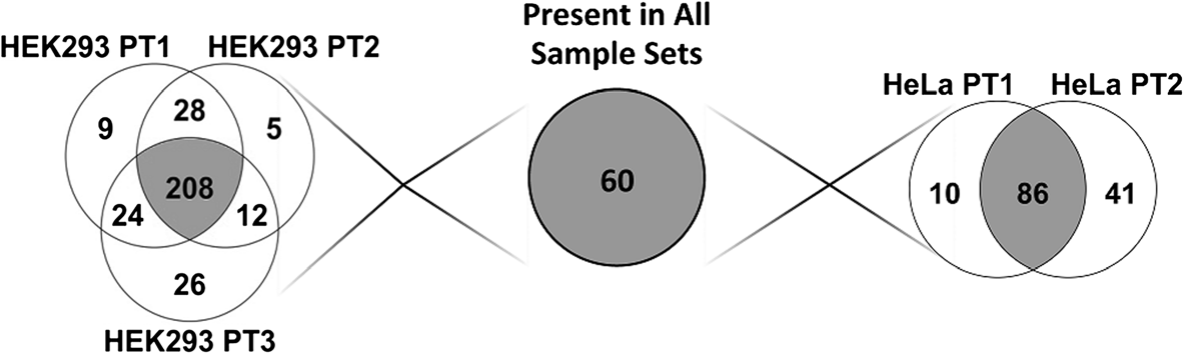
**Replicate experiments using PT from cell lines HEK293 and HeLa cell.** Protein identification of PT elute was achieved with the use of mass spectrometry and analyzed with Scaffold version 3.6.2, Proteome Software Inc. Any protein identified with greater than a 99% possibility and two unique peptides with at least 95% probability were considered. Reproducibility of triplicate PT experiments with HEK293 cell line is shown by the overlapping region of HEK293 PT 1, 2, and 3 with 208 proteins in common. HeLa cell line was also compared in duplicate and was found to have 86 proteins which occur in both experiments. 60 proteins were found to be in common in all five of the experiments. These proteins are specified in an Excel spreadsheet file in Additional file 2: Table S2.

To illustrate the reproducibility of PT, triplicate PT eluates were analyzed from the HEK-293 cell line by mass spectrometry (Figure 9). Replicate experiments of each cell line were compared with Scaffold version 3.6.2 and identifications were accepted with a minimum of 99% protein probability. For HEK293, 208 proteins were found to be in all three purifications. These are shown in Additional file 1: Table S1 as a hyperlinked Excel spreadsheet where more information can be found. Most are either known DNA- or RNA-binding proteins, including known components of the TFII complex. Additionally, TFs purified by PT from HeLa nuclear extract had 86 proteins found in duplicate experiments. Since the same amounts of proteins were analyzed using the same analysis parameters, we conclude that the two cell lines differ in the exact composition of their transcription complex. A further comparison of the two cell lines showed that the pooled HEK-293 results when compared to the pooled HeLa results have 129 proteins in common. Further investigation must be done to dissect the significance of these findings. However, based on MS data acquired for these two cell lines, over 100 proteins are bound by the promoter in a transcriptionally active complex.

**Figure 10:**
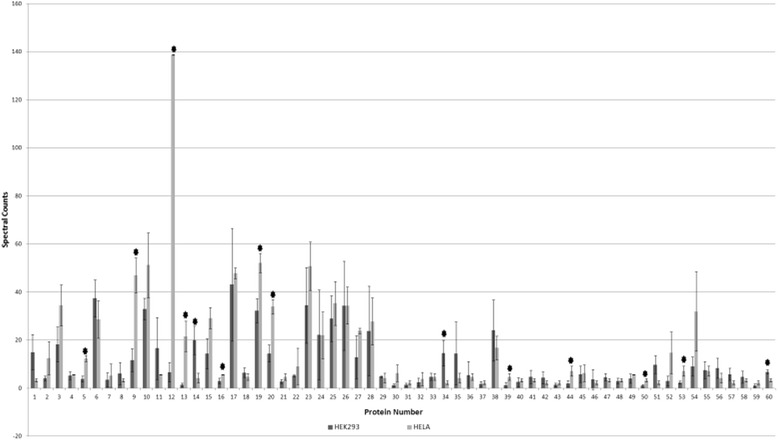
**Graphical representation of proteins identified in all sample sets.** Each promoter trap sample was treated individually to identify key proteins that are present in all data sets. Sixty proteins were found in all of the promoter trap experiments. Resulting proteins were then compared to each other based on cell line with HEK293 shown as black bars and HeLa is shown in grey. The protein numbers (abscissa) are from Additional file 2: Table S2 while spectral counts are shown on the ordinate. Error bars are calculated based on standard deviation. Asterisks above specific proteins corresponds the analysis of variance. A significant difference based on 95% confidence interval calculated by ANOVA is shown for 12 separate proteins.

To analyze the core of the transcriptional complex, each promoter trap experiment was analyzed individually. Sixty proteins were found in each of the five experiments (Additional file 2: Table S2). This analysis eliminates any protein not found in every sample, and since AP-2 and Sp1 were missing from one or more of the data sets, they are not included. Of this set, 52% were found to be involved in transcriptional regulation. The data also shows that 50% are involved in RNA processing and 22% in DNA processing. 28% of the identified proteins are known to be involved in transcription. The 60 proteins were then grouped according to their cell line, to determine if there was a significant difference based on spectral counts, which could implicate regulatory differences amongst different biological sets (Figure 10). The protein numbers on the abscissa are those from Additional file 2: Table S2. Asterisks displayed in the graph correspond to a significant difference in spectral counts (95% confidence interval calculated by ANOVA) between HEK293 and HeLa; only twelve proteins were significantly different in spectral counts although they are present in all samples. It should also be noted that both cell lines, HEK293 and HeLa, follow the same protein abundance trend. This suggests that the core of the transcriptional complex is not dependent on cell type.

In three replicate promoter trap samples using HEK293 we were able to identify transcription factor AP2 however, two were identified as the delta and one identified as the gamma isoforms. SP1 was identified in two of the three samples with one being isoform B. Neither SP1 nor AP2 were identified in HeLa. The mascot scores and percent coverage of AP2 and SP1 for HEK293 are given in Table 1.

**Table 1:** **Identification of hTERT promoter specific transcription factors in HEK293 cell nuclear extract**

	**Percent coverage**	**Mascot score**
Transcription Factor AP2 delta	4%, 4%	28, 33
Transcription Factor AP2 gamma	3%	25
SP1 Transcription Factor Isoform b	2%	25
Transcription Factor SP1	5%	33

From this data we can conclude that promoter specific transcription factors are enriched using the PT technique and detectable through mass spectrometry between 67%-100% for HEK293 but unsuccessful for HeLa cells. One is an embryonic cell (HEK293) while the other is a cancer cell (HeLa) line and this may account for the difference but clearly the method would need further optimization when HeLa cells are analyzed for specific transcription factors. When working with less active promoters such as hTERT instead of the c-jun initially studied, a different approach may be desirable. Success could possibly be increased by using a directed proteomics approach or concentrating the promoter trap elute, assuming that the problem is with a limit of detection and not a lack of enrichment. Here we show that using Promoter Trapping not only can general transcription factors be purified but also promoter specific transcription factorsas well as the ability to visualize the interaction of different transcription factors on each other’s binding site.

## Experimental methods

### Cloning of hTERT-pUC19

50 μL PCR reactions contained 200 nM forward primer (FP, ACG**GGATCC**CTCCCCACGTGGCGGCGGAGG) and reverse primer (RP, CG**GAATTC**GGAGCGCGCGCGCGGCATCGC), 30 μL 1:100 human heart genomic DNA (399 ng total), 200 μM dNTP, 5 μL 10X ThermoPol buffer (New England BioLabs, Ipswich, MA, USA) and 1 U Taq DNA polymerase (New England Biolabs, Ipswich, MA, USA). The bold primer sequences are unique *BamHI* and *EcoRI* sites being added to promoter primer sequences. The mixture was heated to 95° for 5 min and thermocyled 95°C for 1 minute, 60°C for 1 minute, and 72°C for 2 minutes for 35 cycles and finally held at 72°C for 10 minutes for extension. The ~300 base pair product was then gel purified and cloned by ligating EcoRI/BamHI digested fragments into EcoRI/BamHI digested pUC19 vector. The resulting plasmid (hTERT-pUC19) was confirmed to have the hTERT promoter sequence −170 to +91 by DNA sequencing.

### Synthesis of (GT)_5_ tailed hTERT promoter

The tailed hTERT was synthesized in two separate PCR reaction; these differ only in the primers used:

#### Anti-sense reaction

PCR was performed as described above using 200 nM reverse primer (RP), 200 nM 5′ phosphorylated and (AC)_5_ version of forward primer (FP_P_, ACACACACACACG**GGATCC**CTCCCCACGTGGCGGCGGAGG) and 100 ng hTERT-pUC19 as template.

#### Sense reaction

As above except using 5′ phosphorylated and (AC)_5_ version of reverse primer (RP_P_, ACACACACACCG**GAATTC**GGAGCGCGCGCGCGGCATCGC) and forward primer (FP). The results of the two reactions are a DNA fragment in which one stand in each reaction is 5′ phosphorylated and has an (AC)_5_ tail, while the other strand is not phosphorylated and has a 3′ (GT)_5_ tail.

The PCR reactions were purified using the PCR purification kit from Qiagen. The sense reactions and antisense reactions were pooled separately and 200 μL of each pool mixed with 20 μL 10X λ exonuclease buffer (New England Biolabs, Ipswich, MA, USA) and 20 U λ exonuclease, and incubated at 37° for two hours. This allowed only the phosphorylated 5′ end strand to be degraded. The resulting two single strands were gel purified. The sense and anti-sense strands were mixed 1:1 and annealed at 95°C for five minutes and then cooled to room temperature over the course of an hour. To ensure the duplex promoter was formed the digested sense, anti-sense, and annealed DNA were run on a 2% agarose gel. Once the annealed product was confirmed the DNA was concentrated using a 3 kDa MWCO centrifuge filter (Millipore, Billerica, MA, USA) at 4°C. OD_260_ was taken to calculate the concentration using the following equation:

$$ \left(OD260\kern0.5em \mathrm{nm}\right)\kern0.5em \times \kern0.5em \left( dilution\kern0.5em  factor\right)\kern0.5em \times \kern0.5em 50\upmu \mathrm{g}/\mathrm{ml}\kern0.5em =\kern0.5em \mathrm{D}\mathrm{N}\mathrm{A}\left(\upmu \mathrm{g}/\mathrm{ml}\right) $$

### Preparation of HEK293 nuclear extract

HEK 293 cells were cultured and nuclear extract was prepared as described previously by S. Jiang, M.R. Galindo, H.W. Jarrett, Proteomics 10 (2010) 203. HeLa cell were cultured and nuclear extract prepared by the same procedure.

### Preparation of (AC)_5_-Sepharose

(CA)_5_-Sepharose was prepared as described [9].

### Promoter trapping

All operations were performed at 4°. HEK293 nuclear extract (500 μg) and 10 nM duplexed DNA (hTERT promoter), containing a single stranded (GT)_5_ tail on the 3′ ends, were combined in 100 μL 5X binding buffer (BB: 50 mM NaCl, 10 mM HEPES, 10 mM MgCl_2_, 1 mM EDTA, 50 μM ZnSO_4_, 1 mM DTT, pH 7.5 and 30 μg/mL poly dI:dC (Sigma, St. Louis, MO, USA)), in a final volume of 500 μL. The reaction mixture was incubated at room temperature for 30 minutes. The complex is purified by annealing the (GT)_5_ tailed promoter complex to a 1 mL (CA)_5_-Sepharose column. The column is washed with 20 column volumes of binding buffer and then the promoter specific transcription factors are eluted with 5 column volumes of TE0.5 (10 mM Tris, pH 7.5, 1 mM EDTA, 0.5 M NaCl). The eluate was concentrated using an Amicon Ultra-0.5 mL 10 kDa molecular weight cut off centrifuge filter and desalted by buffer exchange with 50 mM ammonium bicarbonate.

### Electrophoresis

Nuclear extract (NE) and promoter-trapped proteins were further fractionated by electrophoresis. Samples were resolved on one dimensional 12% SDS-PAGE (1DE) by the method of Laemmli [14]. In other experiments, two-dimensional electrophoresis (2DGE) was performed with the first dimension being isoelectric focusing, performed on a 7 cm, pH 3–10 IPG strip, and the second dimension further resolves the proteins by their relative molecular mass with the use of a 12% sodium dodecylsulphate-polyacrylamide gel electrophoresis (SDS-PAGE). The resulting SDS-PAGE gel was silver stained to visualize the location of the proteins or characterization of the individual proteins was accomplished with a variety of methods to include mass spectrometry, Southwestern blots, and Western blots.

## Enzymatic digestion

### In-Gel digestion

2DGE gels were cut into 1 mm square blocks. Each gel slice was cut into small pieces and placed into tubes. 200 μL of 100% acetonitrile (ACN) was added to each tube and the gel was allowed to shrink for 10 minutes (acrylamide turns opaque). The supernatant was removed and discarded. 10 mM dithiothreitol (DTT) in 50 mM NH_4_HCO_3_ was added to the gel pieces so that they are completely submerged (~200 μL) and incubated at 37° for one hour and then 500 μL of ACN was added and left for 10 minutes. The supernate was removed and discard. Then 200 μL of 55 mM iodoacetamide in 50 mM NH_4_HCO_3_ was added and the pieces completely submerged and incubated for one hour at 37° in the dark. 500 μL of 100% ACN was next added for 10 minutes. If gel pieces are not opaque, the supernatant is removed and 100% ACN is again added for dehydration. The supernate is again removed and discarded. An excess of trypsin solution (~200 μL, 100 ng/μL of Trypsin Gold, Promega, Madison, WI, USA in 50 mM NH_4_HCO_3_) is added to completely cover gel pieces, allowed to re-hydrate with trypsin on ice or at 4° for 60 min. and then incubated at 37° overnight. To extract the peptides, the tubes are then centrifuged and the supernatant is placed in a fresh tube. 0.1% TFA, 50% ACN was added to the gel pieces and incubated for 15 minutes. The supernatant was then removed and combined with the previous extract. The samples were then dried (SpeedVac) and dissolved in 10 μL 0.1% triflouroacetic acid (TFA).

### Promoter trapping eluate digestion

Urea was added to 100 μL of concentrated promoter trap eluate to a final concentration of 8 M and incubated at 37° for one hour in order to denature the proteins. The sample was made 10 mM DTT by the addition of 500 mM DTT and incubated at 37° for one hour. The protein was alkylated by adding of 400 mM iodoacetamide to the solution to make a final concentration of 40 mM iodoacetamide and incubation at 37° for one hour in the dark. The sample was then diluted 10-fold with 50 mM NH_4_HCO_3_ and 1 μg/μL Trypsin in 50 mM NH_4_HCO_3_ was added to give a final ratio of 1:50 trypsin: protein (w/w). The solution is incubated overnight at 37°. TFA was added to make final concentration 0.1% TFA and the sample applied to a C18 Spin Columns (Pierce, Rockford, IL, USA) and eluted with 0.1% TFA, 70% ACN. Eluate from the C18 Spin Column was further concentrated in a SpeedVac to dryness and then re-suspended in 10 μL 0.1% TFA.

## Characterization

### Mass spectrometry

The resulting peptides were analyzed by capillary LC/MS/MS by injecting 2 μl onto a 50 μm-i.d. column packed to 7 cm of 3 μm C-18 silica and an integrated nano-electrospray emitter with a flow rate of 350 nL/min with a reverse phase gradient of 2 to 62% of 0.1% formic acid in ACN over 60 minutes. Fragmentations of the ten most abundant peptides were carried out with a hybrid linear ion trap-Fourier-transform tandem mass spectrometer (LTQ-Elite, ThermoFisher, San Jose, CA, USA) via high-energy C-trap dissociation in positive ion mode. Multiple charged peptide precursor ions were fragmented to give spectra for the complementary N- and C-terminal sequence-specific product ions.

### Protein database searching

Database searching was carried out using a 10-node Mascot cluster (version 2.3.02, Matrix Science, London, UK) using the Swiss-Prot database (release 2012_11; 538,577 sequences). Search criteria included peak picking with Mascot Distiller, 10 ppm precursor ion mass tolerance, 0.8 Da product ion mass tolerance, three missed cleavages, enzymatic digestion by trypsin, and oxidation of methionine and iodoacetamide derivatives of cysteine were specified as variable modifications. Replicate experiments were compared and analyzed with Scaffold version 3.6.2 (Proteome Software Inc., Portland, OR).

### Western blot

Protein collected from Promoter Trapping of 500 μg nuclear extract was further resolved by 12% SDS-PAGE and electro-blotted onto a polyvinylidene fluoride (PVDF) membrane. The membrane was then blocked with 5% milk, 3% BSA in Tris buffered saline (TBS) for one hour at room temperature. The membrane was probed separately with primary antibodies. The antibodies used were TBP, Pol-II, USF-2, SP1, and β-actin from rabbit (Santa Cruz Biotechnology, Inc., Dallas, TX, USA). Each antibody was used in a 1:100 dilution in 5% BSA in TBS. The membrane was allowed to incubate with the primary antibody overnight at 4°C. The following day the membrane was washed once with TBS and then probed with the secondary antibody (goat anti rabbit-horse radish peroxidase, Santa Cruz Biotechnology, Inc., Dallas, TX, USA) at 1:5000 in 5% milk. Detection was accomplished with enhanced luminol-based chemiluminescent substrate (ImmunoCruz, Santa Cruz Biotechnology, Inc., Santa Cruz, CA, USA) and instructions provided by the manufacturer.

### Southwestern blot

Following the method of [15], the PT elute was separated by 2DGE and then electro-blotted onto a PVDF membrane. Proteins were renatured and the membrane blocked [15]. The next day the membrane was washed four times with Southwestern blot buffer (SWBB: 10 mM HEPES/NaOH, pH 7.9, 50 mM NaCl, 10 mM MgCl_2_, 0.1 mM EDTA, 1 mM DTT, 50 μM ZnSO_4_, and 0.1% Tween) and then incubated with 2 nM radiolabeled hTERT promoter DNA probe in SWBB containing 10 μg/mL poly dI:dC and 0.25% BSA. The next day the membrane was washed with SWBB and exposed to film for 12 hours for autoradiography.

### Electrophoretic Mobility Shift Assay (EMSA) and competition assay

EMSA was performed using ^32^P labeled oligonucleotide probe containing specific oligonucleotides sequences for SP1, AP-2 and E-Box (purchased commercially). DNA-protein complexes were resolved on a non-denaturing 5% polyacrylamide gel and visualization by autoradiography as previously described [16]. Competition assay was accomplished by addition of 40-fold molar excess of unlabeled competitor DNA, SP1, AP-2, E-Box, or hTERT, to the Promoter Trap elute prior to adding radiolabeled oligonucleotide.

### Transfection and luciferase reporter assay

The hTERT promoter DNA was subcloned from pUC19 to pMLUC luciferase vector (Novagen, San Diego, CA, USA) between the BamHI and EcoRI restriction sites. HEK293 cells were plated onto a 12-well plate with 90,000 cells (500 μL) and allowed to incubate at 37° for 24 hours in DMEM medium supplemented with 10% fetal bovine serum resulting in 60% confluence. In two separate microfuge tubes the following were combined to make a 100 μL transfection media and allowed to incubate at room temperature for 15 minutes: 90 μL serum free medium, 2 μg pTK-Luciferase normalization reporter DNA, 5 μL GeneJuice® (EMD Millipore, Billerica, MA) and 0.023 μg of either empty vector control or hTERT-pLUC. After incubation, the medium is removed from the plates and replaced with 500 μL of 10% serum media. 20 μL of the different transfection media was added to separate wells in triplicate and the plate was incubated at 37° overnight. The next day, the media is replaced and incubate at 37° for an additional 24 hours. The cells are then harvested and assayed for firefly and *Renilla* luciferase with the reagents and procedure provided by Dual Luciferase Reporter Assay System (Promega, Madison, WI). Briefly, the media is removed and the cells washed with phosphate buffered saline. 100 μL of passive lysis buffer was added and rocked at room temperature for 30 minutes. 30 μL of the cell lysate was added to 20 μL LARII and placed in a luminometer to take an initial reading. Then, 50 μL of Stop and Glo® Reagent was added and a second reading was taken. The *Renilla* luciferase activity (hTERT) was divided by the Firefly luciferase activity (TK) to give relative luciferase activity.

### Transcription assay

Transcription was measured by a primer extension method. PT elute obtained from 200 μg nuclear extract was diluted to a final volume of 200 μL in TE0.1 buffer in the presence of 10 nM untailed hTERT promoter DNA (final concentration), 600 μM rNTP, 25 units RNasin, 2.5 mM DTT, 3U creatine phosphate kinase, and 12 mM phosphocreatine and incubated for 60 minutes at 30°. The produced RNA was extracted with phenol/chloroform and precipitated with ethanol. For primer extension, the RNA was dissolved in 10 μL annealing buffer (5 mM Tris–HCl, pH 8.3, 1 mM EDTA, and 75 mM KCl) containing 0.1 pmol ^32^P labeled oligonucleotide primer (5′-cggagcgcgcggcatcgcgg-3′) and annealed at 50° for 45 minutes. Primer extension was achieved by adding 20 μL annealing buffer to produce a final solution concentration of 15 mM DTT, 4.5 mM MgCl_2_, 0.5 mM dNTP, 1.5 μg actinomycin D, 25 units RNasin, and 200 U Moloney Murine Leukemia Virus reverse transcriptase to make a final volume of 30 μL and incubated at 37° for 60 minutes. The product was separated with 8 M urea in a 6% polyacrylamide gel and visualized by autoradiography. As a negative control the experiment was repeated without the addition of rNTP or without the addition of hTERT promoter DNA to ensure any visualized bands were not artifacts.

## Additional files

Additional file 1:
**hTERT Promoter Trap Protein Identifications in Cell Line HEK293.** A list comprised of all 208 proteins, with hyperlinks, identified in all three replicate PT experiments. Proteins were chosen based on a minimum of 99% protein probability using Scaffold version 3.6.2. Additional file 2:
**Common Proteins Identified in Cell Lines HEK293 and HeLa using the hTERT Promoter.** A chart comprised of 60 proteins that were identified in both HEK293 and HeLa cell lines. Proteins were chosen based on a minimum of 99% protein probability using Scaffold version 3.6.2. A significant difference in spectral counts, based on a 95% confidence interval calculated by ANOVA, was denoted by an asterisk.

## Abbreviations

2DGE: Two-dimensional gel electrophoresis
2DGE-SW: Two-dimensional Southwestern blot
AP-2: Activator protein 2
E-Box: Enhancer box
FT: Flow through
GTF2-I: General transcription factor II-I
hTERT: Human telomerase reverse transcriptase
NE: Nuclear extract
Pol II: RNA Polymerase II
PT: Promoter trapping
PTE: Promoter trapping eluate
SP1: Specificity protein
TF: Transcription factor
TR-CTCF: Transcription Repressor CTCF
USF: Upstream stimulatory factor
WCL: Whole cell lysate
